# Probability matching is not the default decision making strategy in human and non-human primates

**DOI:** 10.1038/s41598-022-16983-w

**Published:** 2022-07-30

**Authors:** Carmen Saldana, Nicolas Claidière, Joël Fagot, Kenny Smith

**Affiliations:** 1 Department of Comparative Language Science, University of Zurich, Zurich, Switzerland; 2 Center for the Interdisciplinary Study of Language Evolution, University of Zurich, Zurich, Switzerland; 3 Aix-Marseille Université, CNRS, LPC, FED3C, Marseille, France; 4 Station de Primatologie-Celphedia, CNRS UAR846, Rousset, France; 5 Institute for Language, Communication and the Brain, Aix-Marseille Université, Marseille, France; 6 Centre for Language Evolution, The University of Edinburgh, Edinburgh, UK

**Keywords:** Evolution, Psychology

## Abstract

Probability matching has long been taken as a prime example of irrational behaviour in human decision making; however, its nature and uniqueness in the animal world is still much debated. In this paper we report a set of four preregistered experiments testing adult humans and Guinea baboons on matched probability learning tasks, manipulating task complexity (binary or ternary prediction tasks) and reinforcement procedures (with and without corrective feedback). Our findings suggest that probability matching behaviour within primate species is restricted to humans and the simplest possible binary prediction tasks; utility-maximising is seen in more complex tasks for humans as pattern-search becomes more effortful, and we observe it across the board in baboons, altogether suggesting that it is a cognitively less demanding strategy. These results provide further evidence that neither human nor non-human primates default to probability matching; however, unlike other primates, adult humans probability match when the cost of pattern search is low.

## Introduction

Human decisions are not always rational: we do not always make choices that maximise expected utility. A well known example of a putatively irrational behaviour whose nature is still much debated after decades of research is *probability matching*. Probability matching occurs in learning experiments when subjects given probabilistic input respond in a way that is proportional to the observed reward probabilities, or the observed frequencies in paradigms lacking rewards. For example, suppose one has to choose between two alternatives (e.g., two buttons) with different reward probabilities, one with reward on 70% of trials and another with reward on 30% of trials. The optimal decision strategy in this context is to always select the alternative with the highest reward probability, known as *maximising*: on a task with a 70–30 reward ratio, maximising would secure the reward 70% of the time. A participant using a probability matching strategy would instead select the higher-rewarding option roughly 70% of the time and the other option 30% of the time, leading to reward on average only 58% of the time ($0.7^2 + 0.3^2 = 0.58$). A participant using an *over-matching* strategy would fall somewhere between maximising and probability matching, selecting the higher-rewarding option more frequently than its observed 70% reward frequency, but sometimes selecting the other option and thus obtaining an intermediate level of reward.

Despite being sub-optimal, probability matching and over-matching are reported in simple two-alternative (*binary*) prediction tasks with adult human participants^1–3^. These behaviours are not restricted to decision tasks; they also occur in other domains such as in language learning—i.e., when learners encounter “different ways of saying the same thing” in their input, they tend to reproduce those variants at the observed proportions^4–6^. One possible explanation for this behaviour is provided by the *dual-system* account on decision making: probability matching or over-matching are intuitive and readily-accessible response strategies for human participants, providing a spontaneous but incorrect probability matching response which is only replaced by a less-accessible maximising strategy after an effortful deliberative evaluation process^7–9^. The initial intuitive response is thought to result from a bias towards expectation generation, which leads humans to focus on sequence-wide expectations rather than on individual trial outcomes. Consistent with this account, Koehler and James^9^ show that participants are more likely to use a maximising strategy when they are explicitly introduced to it as a possible strategy, and participants who probability match still identify maximising as a more optimal strategy in a post-task questionnaire. However, the sub-optimal behaviour of human adults on these simple tasks is particularly puzzling given that children and non-human animals often maximise in similar tasks: young children appear to exhibit less probability matching behaviour than adult humans^4,10–13^, and early probability learning experiments with non-human animals suggest that mammals such as monkeys^14–20^ or rats^21^ tend to maximise, although other species such as cockroaches, fish or pigeons^21–23^ probability match. Why would a human adult perform on a par with a cockroach and be outperformed by a monkey or a 6 year-old child on such a simple task? Or in dual-system terms, why is the optimal maximising strategy only available to adults after effortful deliberation, but intuitive and readily-accessible to monkeys and small children?

One (reassuring, for adult humans) alternative explanation to the dual-system account is that probability matching reflects the well-known human tendency to see patterns in noise^24^. Sequences where each outcome is randomly and independently drawn (as in a classic probability matching experiment) are rare in life, and adult human decision makers might be particularly strongly biased to believe that sequences follow a pattern, which they try to figure out. Moreover, environments in which probabilities of reward are stationary over time are rare in real life: probability matching could thus result from the misapplication of an otherwise sophisticated adaptive response beneficial in mutable environments^25,26^. The belief that sequences are always pattern-driven and/or that environments are mutable rather than stationary would drive humans to persist in exploring potential pattern-based explanations for their observations, thus producing behaviour that, on average, roughly matches the observed probabilities. This *pattern search* hypothesis is consistent with the observation that participants who use probability matching strategies in the absence of a pattern achieve greater accuracy in the presence of one^27,28^, suggesting that their behaviour in both cases involves a search for patterns. In contrast, participants are more likely to maximise when the outcome sequence in a probability learning task is manipulated in ways which enhance participants’ perception of its randomness^29^, or when they are explicitly told or shown that the outcome sequence is random^30–36^—presumably because these manipulations reduce their propensity to search for patterns. Increasing the complexity of the task by introducing more alternatives also leads to higher rates of maximising behaviour: choices of the higher-rewarding alternative increase in three-alternative (*ternary*) prediction tasks^37^ and in four-alternative prediction tasks^38^ compared to binary prediction tasks, perhaps because it renders pattern search more challenging. Equivalent results are also found in linguistic variation-learning tasks^4^. Also consistent with this hypothesis is the evidence suggesting that adult participants in linguistic variation-learning or sequence-learning tasks tend to introduce context-based conditioning when confronted with random variation^39,40^ whereas children are less prone to do so^41^. Further evidence for the pattern-search hypothesis comes from the neurobiological basis of probability matching behaviour. For example, Miller et al.^42^ found a pattern of brain activation in predictive tasks that differed from a simple detection task (i.e., paying attention to which target had given reward in the last or third-last trial) which was modulated by the participants’ probability matching behaviour: the difference between prediction and detection decreased as participants deviated more from probability matching. However, this pattern search hypothesis is inconsistent with the finding that adult participants still produce probability matching responses in tasks where the opportunity for pattern search is reduced, for example, when presented with aggregated input rather than an input sequence, and when asked to generate aggregate predictions rather than a sequence of predictions^43^.

An alternative *evolutionary* interpretation has also been put forward in the literature. For example, Brennan and Lo^44^ propose that what look like individual sub-optimal behaviours are actually optimal behaviours for population growth under natural selection in certain environments. For example, let us consider an evolutionary scenario where choice *a* makes any individual reproduce with a probability of 0.7, and choice *b* leads to the inverse reproduction rates (i.e., leads to reproduction when *a* does not). Under this scenario, if all individuals maximised their reproductive rates by always choosing *a*, the population would inevitably become extinct when choice *b* were the successful strategy. Although this evolutionary perspective is worth considering when examining the robustness of perceived irrational behaviour in human decision making, its influence on individuals’ behaviour in the type of prediction tasks described so far is not clear^45^. Further, it is important to note that it requires the possibility of a low-rewarding choice at the individual level which could lead to a very high reward at the population level; this possibility is generally absent in probability learning experiments. Moreover, this hypothesis alone does not explain why children and non-human primates are more likely to maximise, and why rates of maximising behaviour might increase with an increment of task complexity (e.g., in a ternary prediction task) where the cost of maximising would not decrease compared to a binary prediction task (with the same probability of reward for the high-rewarding variant).

One possible interpretation of the conflicting results in the literature is that probability matching is not necessarily the product of a single process, but rather the result of a mosaic of mechanisms which produce similar behavioural outcomes even though they might operate in different circumstances^28,46,47^. However, the interpretation of the literature is complicated by the fact that behaviour on probability learning tasks is very sensitive to the details of the task and, for adults at least, its framing^3^. Given this sensitivity to task design and implementation, it is particularly problematic that the methods used often differ across species or age-groups, meaning that directly comparable studies are relatively rare. For instance, experiments with adult humans typically do not feature concrete rewards (getting a trial right is the only reward), whereas experiments with animals and children more often or always feature actual rewards. And to give another example, experiments with humans (particularly children, to a lesser extent adults) tend to be relatively short compared to the long trial sequences provided to animals—this is despite the fact that there is evidence that the presence of rewards and more trials both tend to boost over-matching or maximising behaviours^1,3,16,36,48–51^. Furthermore, potentially informative manipulations have not been systematically applied across species or age-groups, resulting in an incomplete picture of which factors influence behaviour across groups and potentially obscuring commonalities across species and age-groups. For example, manipulating task difficulty using binary or ternary tasks is potentially informative regarding the pattern search account of probability matching, but equivalent results are not available for non-humans. Similarly, the details of the reinforcement procedure affect behaviour in animal experiments: experiments using *guidance* or *correction* procedures where, following incorrect responses, the correct response is highlighted and the animal continues responding until the correct response and the reward is provided, result in less maximising behaviour. This difference is attributed^3,52^ to greater task difficulty in guidance/correction reinforcement paradigms where the trial boundaries are more opaque; however, it could equally be due to a reduced reward for maximising when the reward will eventually be obtained anyway, and the absence of the equivalent manipulations with humans makes this finding difficult to interpret.

In this paper we present a series of four preregistered experiments testing adult humans and Guinea baboons (*Papio papio*) on closely matched probability learning tasks, manipulating task complexity (binary or ternary tasks) and reinforcement procedure (applying either a standard reinforcement procedure where incorrect trials are simply not rewarded, or a guidance procedure where, after incorrect responses, the correct response is highlighted and rewarded when selected). Our findings suggest a refined version of the pattern search hypothesis outlined above: there appears to be a consistent ranking of strategy difficulty across species, with probability matching (resulting from pattern search) being restricted to humans and the simplest possible tasks; maximising is seen in more complex tasks for humans and even in easier tasks for baboons, suggesting that it is a cognitively less demanding strategy; however, baboons (but not humans) will abandon maximising for even less costly alternative strategies (random responding or mostly providing responses at the same location) if the cost of incorrect responses is reduced using a guidance procedure, suggesting that maximising itself is somewhat cognitively costly and those costs could be higher for baboons than humans.

## Results

### Experiment 1: Adult humans probability match or over-match in binary tasks, and maximise in ternary tasks

**Figure 1:**
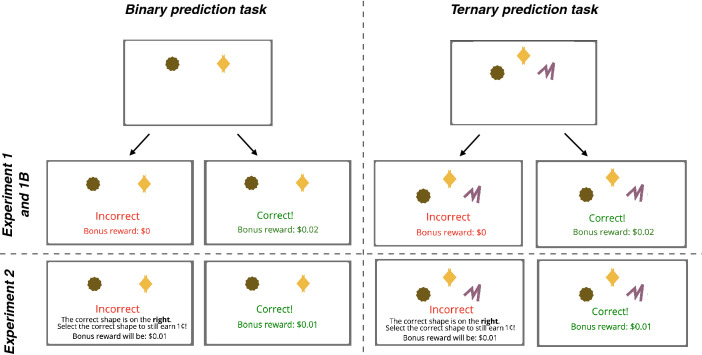
Example test trials for binary (left) and ternary (right) tasks. Across Experiments 1, 1B and 2, adult human participants are presented with shapes and are asked to select one using the arrow keys; in binary tasks, they only use the left and right keys, and in ternary tasks, they use the left, up and right keys. At each trial, only one of the shapes is the assigned target, pooled from the empirical reward distribution for the specified experimental condition. In Experiment 1 and 1B (standard reinforcement procedure), participants receive feedback after selection, which indicates whether their selection is correct or not and the bonus reward accumulated. In Experiment 2 (guidance reinforcement procedure), participants also receive the same feedback on their selection. However, after incorrect responses they are told which would have been the correct response and are asked to select it to be rewarded. In Experiments 3 and 4 we adapt the procedures applied in Experiments 1 (and 1B) and 2 for baboons. Baboons are presented with the same shapes and are asked to select one by screen-touch. In Experiment 3 participants receive a pellet of dry wheat after a correct selection and a green screen signalling failure after an incorrect selection. In Experiment 4 correct responses lead to a food reward (pellet of dry wheat) and incorrect responses are followed immediately by a correction trial (without the display of a green screen). In the correction trial, all shapes disappear from the screen aside from the target shape, and the trial ends when the remaining shape is selected, triggering the delivery of the food reward.

In Experiment 1, human participants took part in a probability learning experiment where, on each trial, they were presented with geometric shapes and had to select one (see Fig. 1); participants were monetarily rewarded for each correct prediction, and different shapes potentially had different probabilities of reward. Participants completed 240 such trials, organised in 4 blocks of 60. We manipulated two factors: the number of alternatives presented on each trial (two shapes in the binary task, three in the ternary task) and the reward distributions (uniform, or skewed). In uniform reward distributions all alternative shapes led to reward with the same probability while in skewed reward distributions, there was a high-rewarding alternative which led to reward 70% of the time, with the remaining reward spread across the remaining choices (i.e. 70-30 in the binary task, 70-15-15 in the ternary task). This led to 4 conditions, which we refer to as Uniform 2, Skewed 2, Uniform 3 and Skewed 3. All factors were manipulated between participants. Further details on the experimental design can be found in “Methods” section.

Our preregistered expectation^53^ was that participants in Experiment 1 will be (1) more likely to maximise when a maximising strategy is available (i.e., in the presence of a higher-rewarding alternative in the Skewed conditions), they will (2) increase their tendency to maximise over time, and they will (3) maximise more in ternary than in binary prediction tasks; indeed, we expected at least some probability matching or over-matching in our Skewed 2 task. Note that in the Uniform conditions, there is no available maximising strategy and we expect participants to probability match; these conditions provide a control for any deviation from probability matching in Skewed conditions.

If participants select a higher-rewarding alternative more often than it was rewarded in their input (thus over-matching or maximising), the variability within their set of responses will be lower that in the actual reward distributions. We quantify this by measuring entropy, a standard measure of variability, and in particular *entropy drop*, the difference between the entropy of the participants’ responses and the entropy of the underlying reward distribution on the task (see “Methods”). Maximising behaviour would lead to maximum entropy drops as the output entropy would be 0 if participants only choose one alternative; probability matching would lead to no difference between the input and output variability, that is, 0 entropy drop; entropy drop greater than 0 but less than the maximum indicates over-matching.

**Figure 2:**
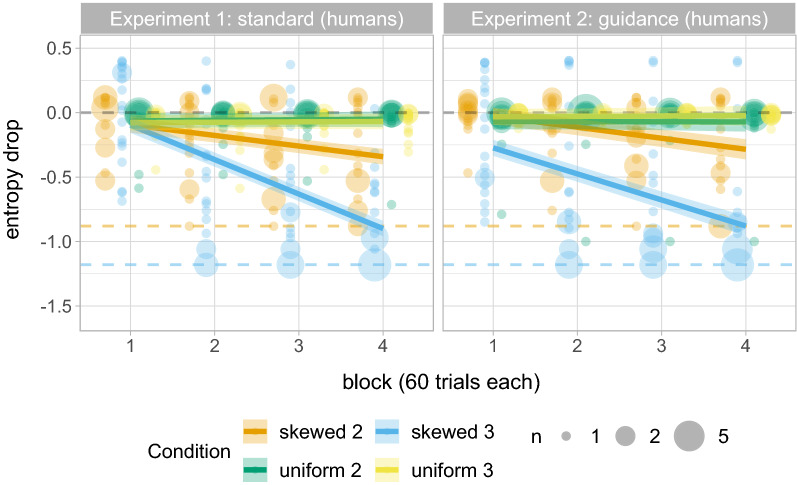
Entropy drop across all four conditions (Uniform 2, Uniform 3, Skewed 2, Skewed 3) in Experiments 1 (standard) and 2 (guidance) with adult human participants. We show the predicted means and standard errors of the fitted models as well as the individual’s raw proportions; bigger dots represent more participants, as specified in the legend. Grey dashed lines represent the input entropy. Coloured dashed lines represent the maximum entropy drop in Skewed 2 (orange) and Skewed 3 (blue) conditions.

A visual inspection of the results (Fig. 2, left) suggests that, in conditions with skewed reward distributions, the entropy of responses decreases by block, although it does not reach the maximum entropy drop within 240 trials. In contrast, entropy drop in conditions with uniform reward distributions stays close to 0. The results from the linear regression model support these observations. We find a higher decrease in entropy in Skewed conditions ($\hat{\beta }=-0.146$, $se=0.027$, $p<0.001$) and in ternary prediction tasks ($\hat{\beta }=-0.074$, $se=0.027$, $p=0.008$); the difference between binary and ternary prediction tasks is nonetheless higher for Skewed conditions ($\hat{\beta }=-0.066$, $se=0.027$, $p=0.016$). Results further suggest a significant decrease in entropy by block ($\hat{\beta }=-0.086$, $se=0.009$, $p<0.001$), and this decrease is significantly greater in Skewed conditions ($\hat{\beta }=-0.089$, $se=0.009$, $p<0.001$) and in ternary tasks ($\hat{\beta }=-0.047$, $se=0.009$, $p<0.001$); a significant interaction between block, probability distribution and number of alternatives further suggests that difference in the per-block entropy drop between binary and ternary tasks is greater in Skewed conditions ($\hat{\beta }=-0.046$, $se=0.009$, $p<0.001$). (Note that we drop the by-participant random slope for the effect of block (as specified in the Analysis section) due to convergence issues (i.e., the random effects correlation is equal to 1 thus suggesting that we might not have enough data to estimate all the parameters reliably). However, the results are the same with or without the random slope.)

**Figure 3:**
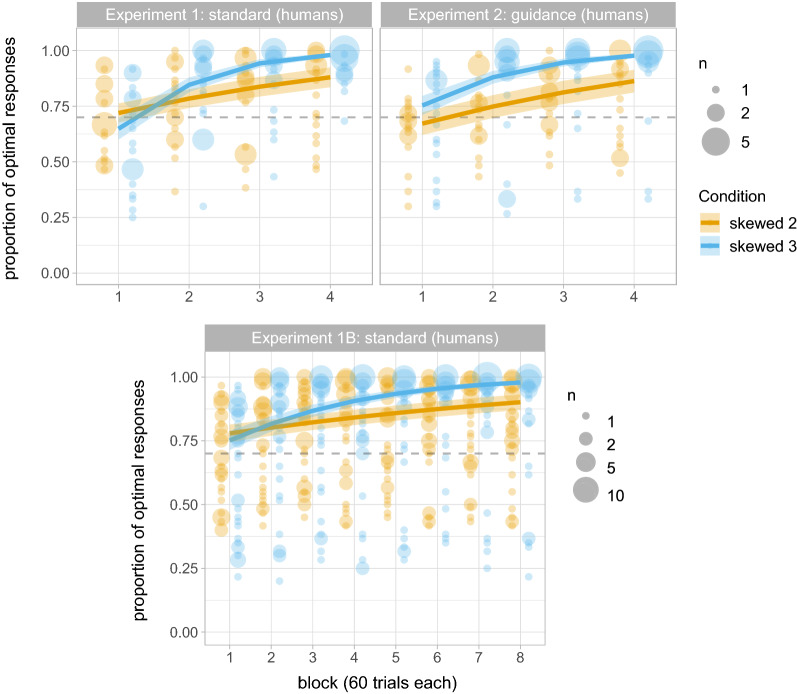
Proportion of optimal responses in conditions with a higher-rewarding alternative (i.e., Skewed 2 and 3) in Experiments 1 (standard), 1B (standard) and 2 (guidance) with adult human participants. We show the predicted means and standard errors of the fitted models as well as the individual data points (colour-faded); bigger dots represent more participants, as specified in the legend. The grey dashed lines represent the input reward probability of the higher-rewarding alternative.

These results are consistent with our predictions: we find larger entropy drops in conditions with skewed reward distributions, and in ternary than in binary tasks. However, this difference between Skewed 2 and Skewed 3 conditions could be explained by the differences in the maximum entropy drop across conditions; since binary and ternary tasks differ in the entropy of their reward distribution, they differ in their maximum entropy drop. Figure 3 (top-left) shows the proportion of optimal responses (i.e. selections of the more frequently-rewarded alternative) in the Skewed conditions. Although participants on average provide the optimal response around 70% of the time in the first block of the experiment, they increase their proportion of optimal responses with every added block of trials ($\hat{\beta }=0.721$, $se=0.089$, $p<0.001$), and are choosing the optimal response significantly above 70% midway through the task ($z =4.971$, $p<0.001$). The increase by block is greater in the ternary task ($\hat{\beta }=0.371$, $se=0.088$, $p<0.001$).

**Figure 4:**
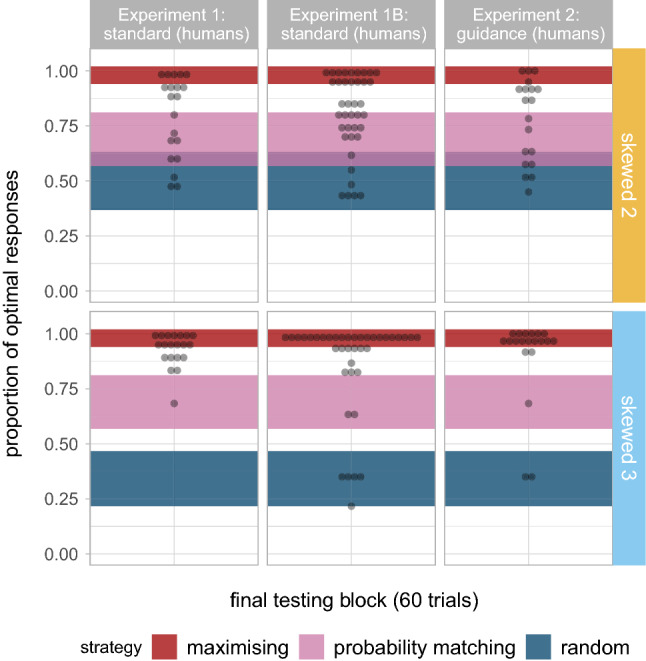
Proportion of optimal responses in the last testing block. Shaded areas signal the binomial proportion confidence intervals for each of the following three strategies: maximising (P = 1), true probability matching (P = 0.7) and random selection (P = 0.5 in binary tasks and P = 0.33 in ternary tasks).

Figure 4 shows participants’ proportion of optimal responses in the final block of testing (last 60 trials) and the corresponding selection strategy. We categorise participants as maximising or probability matching based on their proportion of optimal responses, under the assumption that participants would produce the optimal response with probability 1 under a maximising strategy and probability 0.7 under a (true) probability matching strategy, but allowing for some noise around these values according to the binomial proportion 95% confidence intervals. (For maximising, target P = 1, binomial 95% CI [0.94, 1]; for probability matching, P = 0.7, binomial 95% CI [0.568, 0.812]. Random selection strategies are at P = 0.5 (binomial 95% CI [0.368, 0.632]; NB this overlaps with the probability matching CIs for the binary task) and P = 0.33 (binomial 95% CI [0.217, 0.467]) in binary and ternary tasks respectively. Anything in between probability matching and maximising is classified as over-matching.) Only five (out of 20) participants in the binary task are maximising, six are over-matching (i.e., between probability matching and maximising), and the remaining nine participants fall within the probability matching or random selection regions. In contrast, more than half of the participants (12/20) in the ternary task are maximising, seven are over-matching and only one is probability matching.

Altogether, these results suggest a tendency to over-match (i.e., produce the optimal response more frequently than its reward probability) in adult humans, which is modulated by task: we see mostly maximising strategies in ternary tasks, but the proportion of participants who maximise in binary prediction tasks is low. This is consistent with previous results using similar paradigms. However, as reviewed above, there is evidence from humans and other animals that extending the number of trials eventually leads to higher proportions of maximising behaviour. In order to explore this, we ran Experiment 1B, a preregistered replication^53^ of the Skewed conditions in Experiment 1 with double the amount of trials (8 blocks of 60 trials, 480 trials in total).

Figure 3 (bottom) shows the proportion of optimal responses in Experiment 1B. The model’s results replicate those of Experiment 1: a significant effect of block ($\hat{\beta }=0.262$, $se=0.032$, $p<0.001$) suggests that the proportion of optimal responses increases by block, and the significant interaction with task indicates that optimal responses increase by block more so in ternary than in binary prediction tasks ($\hat{\beta }=0.126$, $se=0.032$, $p<0.001$); there is also a significant difference between tasks by the midway point of the experiment ($\hat{\beta }=0.362$, $se=0.167$, $p=0.030$), which together with the significant interaction between block and task ($\hat{\beta }=0.126$, $se=0.032$, $p<0.001$) further shows that participants in the ternary task do indeed produce higher proportions of the highest-rewarding variant by the end of the experiment.

Figure 4 (middle) shows participants’ proportion of optimal responses in the final block of testing (last 60 trials) in Experiment 1B, along with the corresponding selection strategy. Only 13/39 participants in the binary task are maximising, 8/39 are over-matching, and the rest are either probability matching (11/39) or randomising (6/39), with one further participant ambiguous between probability matching and randomising. In contrast, in the ternary task the majority of the participants are maximising (26/39) or over-matching (6/39), with very few probability matching (2/39) or selecting targets at random (5/39). In other words, doubling the duration of the experiment leads to a very similar distribution of strategies by the end; participants on the binary task do not converge to maximising if given longer on the task. (We ran a further exploratory model predicting the proportion of optimal responses by block and number of variants with an interaction term (as in the previous paragraph) but only including the last four blocks; results confirm that we find not effect of block ($\hat{\beta }=0.109$, $se=0.059$, $p=0.063$) or its interaction with the number of variants ($\hat{\beta }=0.072$, $se=0.056$, $p=0.199$).)

### Experiment 2: A guidance reinforcement procedure does not change human behaviour

In Experiment 2, we use the same design and procedure as in Experiment 1 but use a guidance reinforcement procedure whereby participants were required to correct incorrect selections and subsequently received the reward (see Fig. 1). The inclusion of correction trials eliminates the monetary cost of non-maximising strategies in skewed conditions: participants will get the same overall reward regardless of the strategy they use, and the only penalty for failed trials is the added time (and button click) of the correction trial. If the monetary gain is the main incentive leading to strong over-matching and maximising behaviours in Experiment 1, we expect to observe higher rates of probability matching behaviour across tasks given the reduction in cost; recall also that, as reviewed above, in non-humans this reinforcement procedure leads to reduced rates of maximising (although this seems to be attributed to increased task difficulty in that literature). Moreover, by including correction, we also make sure that the input probabilities of reward are directly observed by the participants in both binary and ternary tasks: in the ternary task using the standard reinforcement procedure from Experiment 1, incorrect response trials leave participants with some residual uncertainty about which of the two responses they *did not* select would have been rewarded on that trial, whereas in binary tasks there is no uncertainty (the response not selected must have been the rewarded response). This difference between binary and ternary tasks could be responsible for the differences between tasks observed in Experiment 1; if so, we expect the guidance procedure to eliminate that difference, since it removes the uncertainty in the ternary condition.

The results of Experiment 2 (Figs. 2, right; 3, top-right) show the same tendencies as in Experiment 1. We found a larger decrease in entropy in Skewed conditions ($\hat{\beta }=-0.146$, $se=0.027$, $p<0.001$) as well as in ternary prediction tasks ($\hat{\beta }=-0.074$, $se=0.027$, $p=0.008$); the difference between binary and ternary prediction tasks is also higher for Skewed conditions ($\hat{\beta }=-0.066$, $se=0.027$, $p<0.016$). There is a significant decrease in entropy by block ($\hat{\beta }=-0.086$, $se=0.009$, $p<0.001$), significantly greater in Skewed conditions ($\hat{\beta }=-0.089$, $se=0.009$, $p<0.001$) and in ternary tasks ($\hat{\beta }=-0.047$, $se=0.009$, $p<0.001$); a significant interaction between block, probability distribution and number of alternatives further suggests that the difference in the per-block entropy drop between binary and ternary tasks is greater in Skewed conditions ($\hat{\beta }=-0.046$, $se=0.009$, $p<0.001$). Looking at proportion of optimal responses (data in Fig. 3, top right), the proportion of optimal responses increases significantly by block ($\hat{\beta }=0.625$, $se=0.082$, $p<0.001$), and more in ternary than in binary tasks ($\hat{\beta }=0.251$, $se=0.081$, $p=0.002$). Looking at the individual proportions of optimal responses in the final block (see Fig. 4), we see the same distribution as in Experiment 1: In the binary task maximising is rare, with many participants over-matching or probability matching; in the ternary task a clear majority of participants are maximising in the final block.

Altogether—and unlike in the literature reviewed for non-humans—we find the same patterns in Experiment 2 with a corrective reinforcement regime as we found in Experiment 1/1B with the standard reinforcement procedure. The results obtained in Experiment 2 thus suggest that the differences between binary and ternary tasks in human participants are not driven by the access to the reward distribution, which was opaque in Experiment 1 (and 1B), but not in Experiment 2. They further suggest that the human tendency to maximise or over-match is not driven (purely) by differences in monetary reward; we do not observe that participants respond at random or explore patterns more in Experiment 2 than in Experiment 1. The results from a further model including Experiment (2 vs 1) and an interaction term confirm this: while this analysis shows the expected effects of block ($\hat{\beta }=0.712$, $se=0.084$, $p < 0.001$) and its interaction with the number of alternatives in the task ($\hat{\beta }=0.367$, $se=0.083$, $p < 0.001$), we find no effect of Experiment or any interactions involving Experiment (smallest $p = 0.102$).

### Experiment 3: Guinea Baboons maximise on both binary and ternary tasks

Experiments 3 adapted Experiments 1 for Guinea baboons (*Papio papio*), using a facility developed by J.F. to allow automated testing of semi-free-ranging baboons using a touch-screen apparatus^54,55^. As with humans, baboons saw a set of coloured shapes at each trial and were prompted to select one (see Fig. 1); each shape lead to a reward according to the ratio specified by the condition (Skewed 2, Skewed 3, Uniform 2, Uniform 3 as in the human experiments; in baboons these manipulations were run within subjects, see “Methods”). If baboons selected the target shape, they were rewarded with a pellet of dry wheat; if they failed to select the target shape, a green screen signalling failure was shown and after a three seconds delay, baboons proceeded to the next trial without reward. For each condition, participants completed 10 blocks of 240 trials each: note that this is longer than for human participants but provides comparable data on blocks 1 and 1-2 for human Experiments 1 and 1B respectively. We extended the duration of the baboon experiment in line with previous work which suggested that differences between standard and guidance reinforcement procedures only emerged in non-humans after many trials (i.e., around 2000)^17,19^.

**Figure 5:**
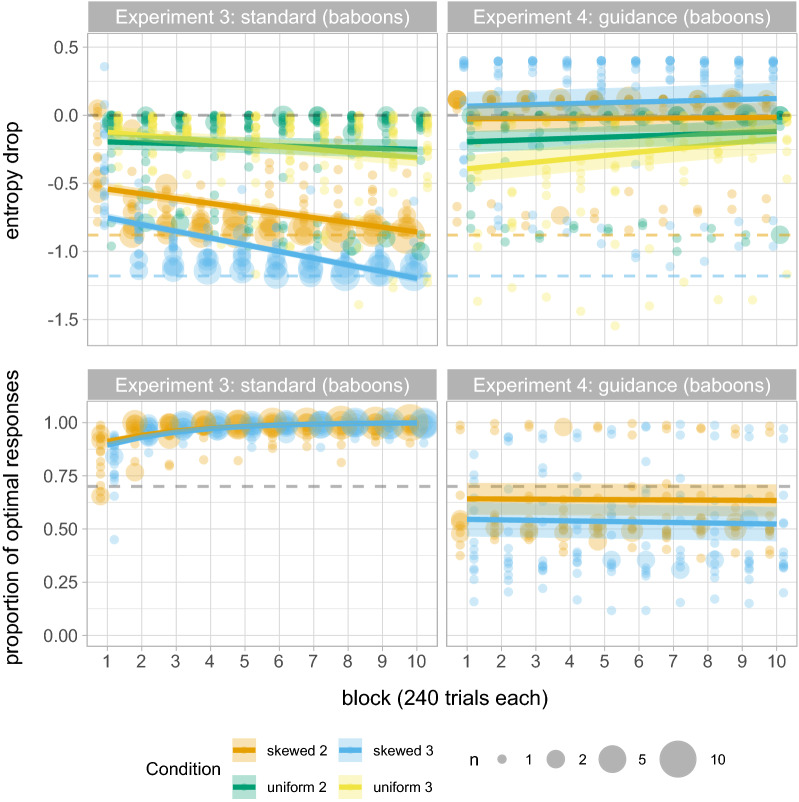
Summary of results for Experiments 3 (standard) and 4 (guidance) with Guinea baboons. Top row: entropy drop across all four conditions (Uniform 2, Uniform 3, Skewed 2, Skewed 3). Bottom row: proportion of optimal responses in conditions with a higher-rewarding alternative (i.e., Skewed 2 and 3). We show the predicted means and standard errors of the fitted models as well as the individual data points (colour-faded); bigger dots represent more participants, as specified in the legend. The grey dashed lines represent the input entropy and the input reward probability of the higher-rewarding alternative. Coloured dashed lines in the top row plots represent the maximum entropy drop in Skewed 2 (orange) and Skewed 3 (blue) conditions.

The results (Fig. 5, top left) suggests a significant drop in entropy in participants’ by the mid-way point of the experiment ($\hat{\beta }=-0.530$, $se=0.031$, $p<0.001$). The entropy drop is more pronounced in conditions with skewed reward distributions where maximising is possible ($\hat{\beta }=-0.308$, $se=0.026$, $p<0.001$), and in ternary tasks ($\hat{\beta }=-0.068$, $se=0.016$, $p<0.001$); this difference between ternary and binary tasks is most distinct in Skewed conditions ($\hat{\beta }=-0.070$, $se=0.006$, $p<0.001$). We also found a significant decrease in entropy by block ($\hat{\beta }=-0.028$, $se=0.004$, $p<0.001$), more pronounced in skewed distributions ($\hat{\beta }=-0.014$, $se=0.002$, $p<0.001$) and in ternary tasks ($\hat{\beta }=-0.007$, $se=0.002$, $p=0.001$). We found no difference in the rate of entropy drop over time between ternary and binary tasks in Skewed conditions ($\hat{\beta }= 0$, $se=0.002$, $p=0.969$). Altogether, these results suggest that entropy drop is larger in skewed than uniform distributions, and in ternary tasks than binary; however, this difference between binary and ternary could be simply explained by the differences in the maximum entropy drop across conditions (see Fig. 5, top left).

**Figure 6:**
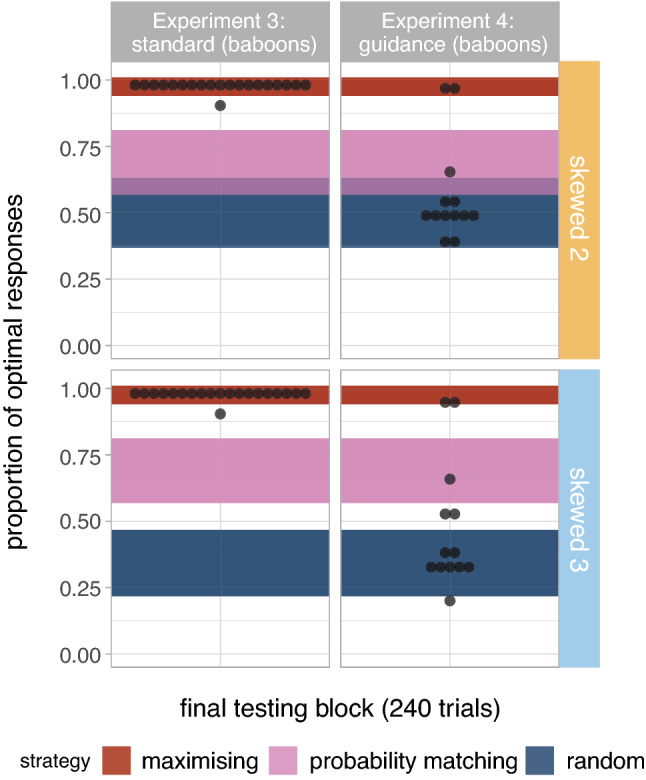
Proportion of optimal responses in the last testing block. Shaded areas signal the binomial proportion confidence intervals for each of the following three strategies: maximising (P = 1), true probability matching (P = 0.7) and random selection (P = 0.5 in binary tasks and P = 0.33 in ternary tasks).

Figure 5 (bottom left) shows the proportion of optimal responses in binary and ternary task conditions with skewed reward distributions. The intercept of the logistic regression model fitted to this data ($\hat{\beta }=4.261$, $se=0.206$) suggest that baboons choose the optimal responses significantly above 70% ($z = 16.571$, $p < 0.001$), and even above 95% ($z = 6.391$, $p < 0.001$), thus confirming that participants fully maximise by the midway point of the experiment, and comparably across binary and ternary tasks ($\hat{\beta }=-0.030$, $se=0.102$, $p=0.769$). The significant effect of block ($\hat{\beta }=0.451$, $se=0.049$, $p<0.001$) shows that the proportion of optimal responses increases over time, at approximately the same rate on binary and ternary tasks ($\hat{\beta }=0.015$, $se=0.008$, $p=0.083$). Figure 6 (left) shows that all baboons use maximising strategies equally across tasks in the final block of testing (last 240 trials). These results thus confirm that baboons overwhelmingly use maximising strategies—when available, i.e., in Skewed conditions—in both binary and ternary tasks (i.e., unlike humans, they do not show more probability matching in the binary task); the difference we found with entropy drop between binary and ternary tasks was not driven by differences in maximising behaviour, but only by the differences in the maximum entropy drop.

### Experiment 4: A guidance reinforcement procedure leads to random responding in Guinea baboons

Experiment 4 once again features baboons, but incorporates the guidance reinforcement procedure used with humans in Experiment 2: in Experiment 4, incorrect responses were followed immediately by a correction trial (without the display of a green screen signalling failure, see Fig. 1); in the correction trial, all shapes disappeared from the screen aside from the target shape, and the trial would end when baboons selected the shape and received reinforcement. Recall that for humans, adding this guidance procedure did not change behaviour. In contrast, results from the skewed conditions in Experiment 4 with baboons show completely different tendencies to those seen in the standard reinforcement paradigm of Experiment 3: we do not observe a decrease in entropy by block in skewed conditions (see Fig. 5, top right), and entropy drop is on average very close to 0 across testing blocks in all conditions, regardless of the number of alternatives in the task or the distribution of reward. (Full details of a linear mixed effects model supporting these observations can be found in the supplementary materials, see Data Accessibility.) The data thus suggest that baboons are not using maximising strategies when they do not lead to higher eventual reward, even though maximising would remove the need to complete the correction trials. The data on the proportion of optimal responses in conditions with skewed reward distributions confirm this (see Fig. 5, bottom right). The model’s intercept suggests that participants’ proportions of optimal responses are not different from P = 0.5 ($\hat{\beta }=0.351$, $se=0.325$, $p=0.279$); consistent with the lower chance level of ternary tasks, the proportion of optimal responses is significantly lower in ternary tasks ($\hat{\beta }=-0.214$, $se=0.009$, $p<0.001$). Unlike in the previous experiment, we did not find a positive effect of block, and we did not find any effect of its interaction with the number of alternatives in the task, suggesting that participants do not increase the proportion of optimal responses over time in either binary or ternary tasks. (Note that we had to drop by-participant random slopes (as specified in the Analysis section) due to convergence errors. However, we found no evidence for a positive effect of block or its interaction with the number of variants neither in the over-fitted model with random slopes; full details of the model can be found in the supplementary materials, see Data Accessibility.)

**Figure 7:**
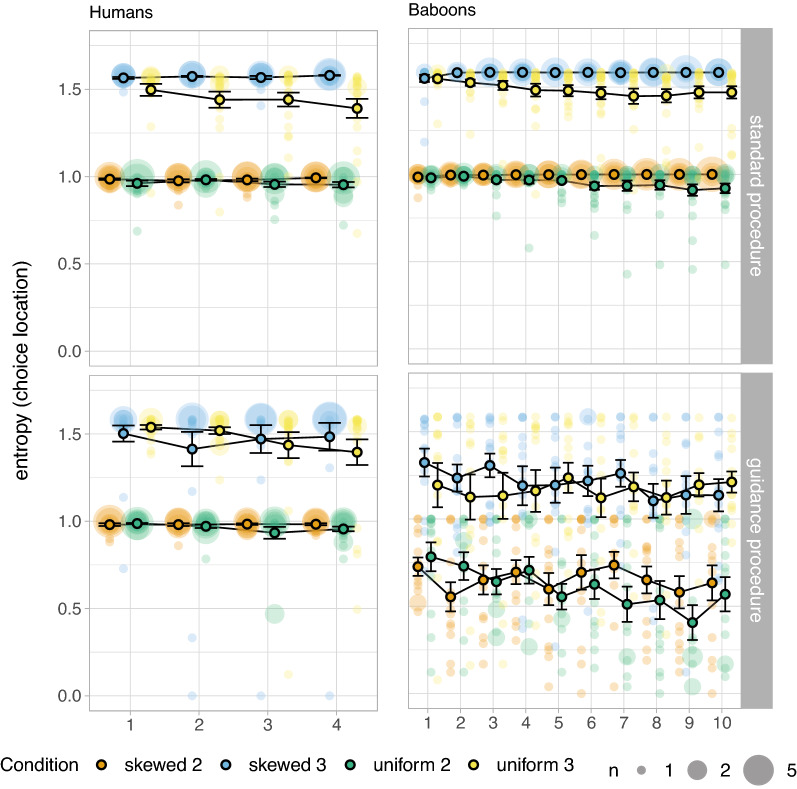
Entropy of the distribution of choice location on screen by block across conditions and species. We show the means and standard errors as well as the single individual data points (colour-faded); bigger dots represent more participants, as specified in the legend.

Looking in detail at the final block of 240 trials in skewed conditions (see Fig. 6, right panels), we observe that only a small minority of baboons (2/13) seem to be using maximising strategies, and the majority seem to be selecting targets randomly. However, this seemingly random strategy is driven by an alternative efficient strategy of fixating responses on a specific location. Figure 7 shows the entropy of the distribution of choice location on screen per block (i.e., left or right in binary tasks; left, centre or right in ternary tasks). Baboons seem to only pivot towards the strategy of choosing the same location on screen across conditions in Experiment 4, and not in Experiment 3 where reward is not always granted; humans, on the other hand, do not seem to converge on this alternative strategy in either experiment.

### Cross-species comparison of maximising behaviour

**Figure 8:**
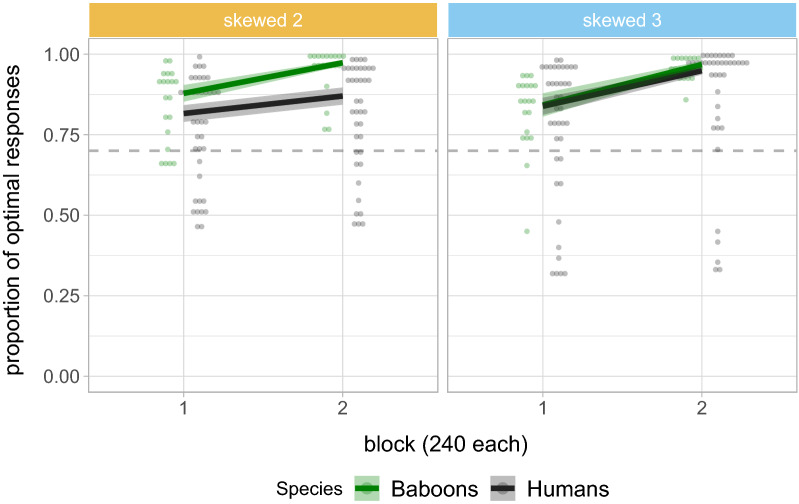
Cross-species comparison of the proportion of optimal responses in conditions with a higher-rewarding alternative (i.e., Skewed 2 and 3). For the baboon data, we used the first two blocks of Experiment 3. For humans, we show the data from Experiment 1B across two blocks of 240 trials each to make it comparable to the blocks of Experiment 3 with baboons. We show the predicted means and standard errors from the fitted models as well as the single individual data points (colour-faded). The grey dashed lines represent the input reward probability of the higher-rewarding alternative.

We further tested the differences in the proportion of optimal responses across species in the standard reinforcement regime with skewed reward distributions (see Fig. 8) . The results from the logistic regression model suggest that participants choose the optimal responses significantly above 70% in ternary tasks (intercept: $\hat{\beta }=2.410$, $se=0.178$; $z = 8.922$, $p < 0.001$). They further suggest that the proportion of optimal responses is comparable for ternary tasks in humans and baboons ($\hat{\beta }=-0.130$, $se=0.178$, $p=0.464$); however, a significant interaction between number of alternatives and species suggests that human participants select the proportion of optimal responses less often than baboons in binary than in ternary tasks ($\hat{\beta }=-0.415$, $se=0.216$, $p=0.055$). We further found a significant effect of block ($\hat{\beta }=1.502$, $se=0.148$, $p<0.001$), suggesting that the proportion of optimal responses increases over time. This increase is comparable across species in ternary tasks ($\hat{\beta }=-0.231$, $se=0.147$, $p=0.116$). In binary tasks, the increase is generally less pronounced ($\hat{\beta }=-0.486$, $se=0.136$, $p<0.001$), but a significant three-way interaction between block, species and number of alternatives suggests that the difference in the proportion of optimal responses across blocks between ternary and binary tasks is greater in humans ($\hat{\beta }=-0.372$, $se=0.135$, $p=0.006$). Altogether, the results suggest that humans’ maximising behaviour in the ternary task is comparable to that of baboons (in both binary and ternary tasks), but humans are less likely to adopt maximising strategies in binary tasks.

## Discussion

Our results suggest tight restrictions on probability matching in human decision making^1^: our participants only consistently probability match in conditions with uniform reward distributions, where all choices are rewarded with the same probability and thus the same overall gain can be obtained regardless of the strategy played. In conditions with skewed reward distributions, where a maximising strategy was available, we observe a significant decrease in the variability of the participants’ productions over testing blocks which corresponds to an increase in the proportion of optimal responses. However, the degree to which participants eventually play maximising strategies is mediated by the task complexity, whether it is binary or ternary (in line with older studies)^37,38^. In ternary prediction tasks, the majority of participants ended up using a maximising strategy. In binary prediction tasks, participants were less likely to maximise, and we saw substantial proportions of participants over-matching, probability matching, or indeed randomising.

Contrary to findings with non-human animals, these results were independent of the reinforcement regime in humans: we found the same tendencies in Experiments 1 and 2. Therefore, monetary cost (at least of the magnitude used in our study) does not seem to be the main driver behind the increase in maximising behaviour over time. Importantly, finding the same difference between binary and ternary tasks even with correction trials allows us to conclude that that asymmetry is not driven by the difference in the transparency of the reward distribution: the guidance procedure ensures that it is explicit on each trial which alternative was the correct one, but the effect of task on maximising behaviour (more maximising in ternary tasks) remained.

In contrast, Guinea baboons reliably converge on maximising strategies when a maximising strategy is available and there is a cost to playing any other alternative strategy; in both the binary and ternary skewed conditions in Experiment 3, where there was a high-rewarding alternative and we implemented standard reinforcement procedure, errors resulted in no reward. Baboons showed an initial exploratory behaviour resembling probability matching followed by a switch to maximising behaviour by the second block of 240 trials. However, when maximising was not necessary to eventually obtain the reward (under the guidance procedure in Experiment 4), most baboons abandoned maximising and instead adopted an alternative efficient strategy of making their selection on the same location on the screen on both binary and ternary tasks, resulting in what superficially looks like a random selection strategy (instead of probability matching or maximising) for thousands of trials.

What can the similarities and differences across tasks and species tell us? Our results are consistent with the pattern search hypothesis, that is, that probability matching in humans (unlike in other species) is driven by the well-attested human drive for pattern search^27,28^. Our results confirm that this bias is not shared with our model non-human primate species, since baboons maximise when maximising strategies are available; this also provides further evidence against a positive relationship between enhanced cognitive abilities (or “smartness”) and maximising in primates^1,2,36,56^. Probability matching behaviour resulting from pattern search is cognitively costly and maximising might be the result of abandoning such a taxing strategy. The differences across binary and ternary tasks we found in humans indicate that the drive for pattern search is indeed mediated by how effortful such pattern search is. Discovering a pattern in ternary tasks is more taxing: remembering the observed sequences involves a higher memory load and there are more potential pattern combinations to consider. Our results suggest that adult humans drop (or more rapidly drop) the exploration of potential patterns when it becomes more costly (i.e., in ternary tasks), shifting to a less effortful (in the long run) maximisation strategy instead. Note that in ternary tasks there is also a slightly higher monetary cost to not maximising, since the reward ratio of the rewarded to unrewarded responses is higher; however, this is unlikely to explain the difference between binary and ternary tasks since we do not observe any drop in maximising behaviour in Experiment 2 where the guidance procedure eliminates that cost.

Our results further suggest that maximising is itself an effortful strategy. Guinea baboons maximise when required to do so to obtain the reward (i.e., under the standard reinforcement regime), but switch to the less effortful strategy of responding randomly or frequently hitting the same location on screen when the main benefit of maximising is removed as in Experiment 4, where the reward will eventually be obtained regardless of their initial prediction. The location of the shapes on the screen is balanced within blocks and thus a tendency to hit the same screen location leads to behaviour that looks like random responding. By using such a strategy baboons actually pay a cost to avoid maximising in Experiment 4, even under the guidance procedure, because they are forced to complete many additional correction trials, and random responding reduces the ratio of actions to rewards (as it takes two touches rather than one to obtain the reward). However, this cost can be minimised by reducing the time taken when selecting on the first screen, which they do if they do not pay attention to the shape information and hit a random response option or the same location on the screen. This suggests that maximising (i.e., tracking the most reliably rewarded response and selecting it on all trials) is effortful for baboons; in contrast, humans persist in maximising on ternary tasks even with the guidance reinforcement procedure. This suggests a continuum of strategy difficulty in primates: probability matching (underpinned by pattern search) being most effortful, with maximising as a less effortful alternative, and random responding (i.e., resulting from the selection of the same location on screen) as the least effortful. Only humans attempt probability matching in the presence of maximising alternatives and even then, only on the easiest binary tasks; baboons back off from maximising when they can respond randomly and still eventually obtain the reward. This pattern of results is also consistent with the observation that very young children often respond randomly^57,58^ or maximise more than older children^59^, that older children are more prone to maximising than adults, but can be made to probability match by reducing task difficulty^10,37,38^, and that non-human animals take longer to reach maximising strategies when the task is made more difficult by using shapes rather than, for example, locations^60,61^.

## Methods

### Experiments with human participants (1, 1B, 2)

#### Experimental design

##### Participants

We recruited 238 participants (Experiment 1: 80 participants, 20 per condition; Experiment 1B: 78 participants, 39 per condition; Experiment 2: 80 participants, 20 per condition) through Amazon Mechanical Turk for a 10-min long experimental session. Participants were over 18 years old, all based in the US and with a percentage of approved assignments $$\ge 95\%$$ (on at least 100 HITs). Participants had a maximium of 50 min to complete the experiment once they accepted the HIT, and had to successfully complete a series of bot-screening questions to start the experiment. There were no further requirements; no participants were excluded. Participants were paid a base rate of $2 plus they received a bonus for each correct response provided as outlined below. We obtained informed consent from all participants.

##### Procedure

On each trial, participants saw a set of coloured shapes (two or three shapes) and were prompted to select one. Each shape lead to a reward according to the ratio specified by the condition—70:30 (Skewed 2), 70:15:15 (Skewed 3), 50:50 (Uniform 2) or 33:33:33 (Uniform 3). If the participant selected the target shape for a given trial, positive feedback text was displayed in green and they were rewarded with a bonus. If the target shape was not selected, negative text was displayed in red and participants proceeded to the next trial without reward (in Experiments 1/1B), or to a correction trial (in Experiment 2). In the correction trial, participants were told which response would have been correct and they were asked to select it to be rewarded. The bonus per trial was $0.02 (in Experiments 1 and 1B) or $0.01 (in Experiment 2, where they were always rewarded). Participants completed multiple blocks of 60 trials each (4 blocks in Experiments 1 and 2; 8 blocks in Experiment 1B); the reward ratios were constant across blocks. In Experiment 1 and 1B the bonus was $0.02; a maximising strategy would thus lead to an overall bonus reward of $3.36 ($$0.7 \times 0.02 \times 240$$ trials) and $6.72 ($$0.7 \times 0.02 \times 480$$ trials) in Experiments 1 and 1B respectively. In Experiment 2 the bonus reward per trial was $0.01, and the overall maximising bonus reward was $2.4 ($$1 \times 0.01 \times 240$$ trials).

Experiment 1 used the standard reinforcement procedure where correct responses were rewarded and incorrect responses were not rewarded. In Experiment 2 we implemented a post-trial guidance procedure. Participants in Experiment 2 were given the possibility to always be rewarded regardless of their initial response: after failed responses, participants were told what the correct response would have been and were asked to select it to be rewarded.

##### Instructions

Previous research has accentuated the role that instructions play on adult human’s responses to prediction tasks: participants are more likely to maximise with instructions that emphasise single-trial predictions^46,62^, and more likely to probability match with instructions that emphasise sequence-based predictions^62^. These results suggest that the difference between human and non-human behaviour might lie in the fact that humans default to sequence-based expectations while other animals are more likely to make trial-by-trial predictions. In the current paper, we want to be able to test adult humans and Guinea baboons in tasks matching as closely as possible. In order to achieve this, we primed human participants to provide trial-based responses in the instructions at the beginning of the experiment. The task instructions were as follows: “For each trial in this experiment, you will be presented with [a pair of / three] abstract shapes. You must choose one of the shapes by pressing either the left or right arrow key that correspond with the left or right image respectively. Your task is to select the correct shape. For each correct answer, you will get a bonus of $0.02 which will add to your payment”.

#### Analysis

In Experiment 1, we wanted to test (1) whether subjects would eventually maximise in the presence of a skewed reward distribution (i.e., where a maximising strategy is available) and (2) whether they would do so more in the ternary rather than binary tasks. With the inclusion of our exploratory Experiment 2, we further wanted to test the same hypotheses with a guidance reinforcement regime, where the cost of probability matching is reduced, and the access to the reward distribution is equal in binary and ternary tasks (i.e. participants see what response would have been rewarded regardless of their initial selection).

As per the preregistered analyses, we analysed the difference between the entropy of the set of shapes selected by participants (output entropy) and the entropy of the set of rewarding shapes (input entropy)^6^. The entropy of the set of output responses *S* is given by $H(S) =-\sum \nolimits _{i=1}^n{P(s_i) log_2 P(s_i)}$,where the sum is over the alternative shapes, and $P(s_i)$ is the frequency of shape $s_i$ in the set of responses, *S*. The entropy of the set of rewarding alternatives in the input is calculated in the same way, but $P(s_i)$ is the frequency of shape $s_i$ in the set of input rewarding alternatives. We refer to the difference between output and input entropy (i.e., the output entropy minus the input entropy) as *entropy drop* because we expect the output entropy to be lower than the input entropy if participants over-match or maximise. If participants select the higher-rewarding shape more often than its probability of reward, the variability of the set of responses will decrease, lowering its entropy. Entropy drop allows us to compare the results from skewed and uniform reward probability distributions; in uniform distributions, where there is no high-rewarding shape, we expect the variability of the set of responses to be roughly the same as the variability in the input.

Crucially, for conditions with skewed distributions of reward we also analysed the choice of the optimal response (whether or not subjects choose the shape with the highest reward probability), which allowed us to assess whether participant are using true probability matching, over-matching, or maximising strategies.

We test differences in entropy drop across conditions (Uniform 2, Uniform 3, Skewed 2 and Skewed 3) using a linear mixed-effects model, with number of alternatives (ternary vs binary tasks), probability distribution of reward (skewed vs. uniform), and block (centered) as fixed effect. We include random intercepts for participant and (when possible) by-participant random slopes for the effect of block. The proportion of optimal responses is compared across conditions (Skewed 2 and Skewed 3) using a logistic mixed-effects model with number of alternatives and block as fixed effects, and by-participant random intercepts as well as slopes for the effect of block. Categorical effects are simple coded: levels are compared to each other directly (with baselines: binary tasks and uniform distributions) and the intercept represents the grand mean across levels. Further details of the models can be found in the supplementary materials (see Data Accessibility).

### Experiments 3 and 4: Guinea baboons

#### Experimental design

Experiments 3 and 4 adapted Experiments 1 and 2 respectively to Guinea baboons (*Papio papio*), using a facility developed by J.F. to allow automated testing of a semi-free-ranging baboons using a touch-screen apparatus^54,55^. The design was as per Experiments 1 and 2 modulo the implementation of a within-participants design and the increase of the total number of trials to 2400—in order to surpass the number of trials in the existing experiments comparing standard and guidance procedures, i.e., 1920 trials^17,19^. The order in which participants carried out the experiments was fixed; only participants that completed all conditions in Experiment 3 would move onto Experiment 4. All shapes were different across conditions and experiments.

##### Participants

The participants for this study were Guinea baboons belonging to a large social group (of 25) at the CNRS Primate Center in Rousset-sur-Arc (France). The baboons live in an outdoor enclosure (700 m$^{2}$) connected to 10 computerised testing booths; baboons have free access to all of them at all times. The identification of baboons at the testing booths is made possible by the two bio-compatible 1.2 by 0.2 cm RFID microchips which baboons have implanted on their forearm. Baboons could thus participate in the experiments whenever they chose, and they did not need to be captured to participate. The experimental software allows an independent test regime for each baboon, irrespective of the test booth. Pellets of dry wheat are used as reward. Baboons were neither water- nor food-deprived.

Participants were required to complete all conditions for Experiment 3 within four weeks, and a maximum of a week per condition. Following this criterion, we excluded the data from five baboons across conditions in Experiment 3 (final $N = 20$). Participants were four males (median age 5 years, min = 3, max = 12) and 16 females (median age 9 years, min = 1.5, max = 24). Only 13 out of these 20 further completed all conditions for Experiment 4 (within the overall time of six weeks allocated for both experiments): these were four males (median age 5 years, min = 3, max = 12) and 9 females (median age 9 years, min = 3, max = 14). (One animal did not complete the last 54 trials (out of 2400) in one of the four conditions in Experiment 4; we included their data nonetheless given that we had enough to compare across blocks and conditions.) It is also worth noting that baboons have no prior experience with prediction tasks of the sort we test in this paper.

##### Procedure

At each trial, Baboons saw a set of coloured shapes and were prompted to select one. Each shape lead to a reward in accordance with the ratio specified by the given condition: 70:30 (Skewed 2), 70:15:15 (Skewed 3), 50:50 (Uniform 2) or 33:33:33 (Uniform 3). Baboons were rewarded with a pellet of dry wheat if they selected the target shape. If baboons failed to select the target shape instead, in Experiment 3 they were shown a green screen signalling failure, and after a 3 s delay, they proceeded to the next trial without reward. In Experiment 4, after a failed selection, participants moved directly onto a correction trial (without the display of a green screen signalling failure); in the correction trial, all shapes disappeared from the screen aside from the target shape, and the trial would end when baboons selected the shape and received the food reward. Baboons completed 10 blocks of 240 trials for each condition; the reward ratios were constant across blocks within a given condition.

#### Analysis

We used the same models as per Experiments 1 and 2 but given the within-participant design, we added by-participant slopes for all fixed effects; random slopes are dropped (as specified in the main text) if models containing them fail to converge. We further ran cross-species analyses for the proportion of optimal responses in conditions with skewed reward distributions. We used the baboon data from the first two blocks of Experiment 3 and the human data from Experiment 1B (which contains 480 trials, and allows us to straightforwardly compare two blocks of 240 trials across-species). We ran a logistic mixed-effects model with number of alternatives (binary or ternary tasks), testing block (centered), and species (humans or baboons) as fixed effects with interaction terms; as random effects, we included by-participant random intercepts and by-participant slopes for the effects of block and number of alternatives. The categorical effect of Species was simple coded so levels are compared to each other directly (humans vs baboons, where the reference level were baboons) but the intercept is the grand mean across levels. The categorical effect of the number of alternatives (binary vs ternary tasks) was treatment coded so we could isolate the effect of the ternary tasks, and directly compare binary to ternary tasks (where the reference level was the latter).

### Ethical approval

The study with baboons received approval from the Animal Welfare and Ethical Review Body of the University of Edinburgh (Ref # OS5-19), and the French Ministère de l’Education Nationale et de la Recherche (approval # APAFIS-2717-2015111708173794-V3). The studies with humans were approved by the PPLS Research Ethics Committee at the University of Edinburgh (Ref # 378-1819/1). All experiments were performed in accordance with the Animals (Scientific Procedures) Act 1986, and APA (7th Ed.) guidelines respectively.

## Data accessibility

All data reported are available at osf.io/qnm57/, as well as the preregistered designs and analysis plans^53^. The preregistered design and analysis plan for Experiment 1 and 2 is accessible at osf.io/b3nke; the preregistered design and analysis plan for Experiment 1B is accessible at osf.io/ky7z8; and the preregistered design and analysis plan for Experiment 3 and 4 is accessible at osf.io/evxk4. The analysis scripts used for the analyses and figures reported are also available at osf.io/qnm57/, as well as all the materials used for the human experiments. Human experiments were coded using the *jsPsych* JavaScript library^63^. Experiments with baboons were coded using a custom software developed by Joël Fagot. All data were analysed using *lme4* and the *tidyverse* collection of R packages^64,65^.
